# The Influence of the Substrate on the Functionality of Spin Crossover Molecular Materials

**DOI:** 10.3390/molecules28093735

**Published:** 2023-04-26

**Authors:** Saeed Yazdani, Jared Phillips, Thilini K. Ekanayaka, Ruihua Cheng, Peter A. Dowben

**Affiliations:** 1 Department of Physics, Indiana University-Purdue University Indianapolis, Indianapolis, IN 46202, USA; syazdani@iupui.edu (S.Y.); japphill@iu.edu (J.P.); 2 Department of Physics and Astronomy, Jorgensen Hall, University of Nebraska, Lincoln, NE 68588-0299, USA; 888tke405@gmail.com

**Keywords:** spin crossover molecules, interfaces, thin films, molecular-based devices

## Abstract

Spin crossover complexes are a route toward designing molecular devices with a facile readout due to the change in conductance that accompanies the change in spin state. Because substrate effects are important for any molecular device, there are increased efforts to characterize the influence of the substrate on the spin state transition. Several classes of spin crossover molecules deposited on different types of surface, including metallic and non-metallic substrates, are comprehensively reviewed here. While some non-metallic substrates like graphite seem to be promising from experimental measurements, theoretical and experimental studies indicate that 2D semiconductor surfaces will have minimum interaction with spin crossover molecules. Most metallic substrates, such as Au and Cu, tend to suppress changes in spin state and affect the spin state switching process due to the interaction at the molecule–substrate interface that lock spin crossover molecules in a particular spin state or mixed spin state. Of course, the influence of the substrate on a spin crossover thin film depends on the molecular film thickness and perhaps the method used to deposit the molecular film.

## 1. Introduction

The development of a molecular-based device is by no means a simple endeavor. While a typical device will consist of a thin film of molecular material, there is the question of how that material will interact with the chosen substrate it is deposited on. Furthermore, the device will likely be attached with electrodes of some kind, in addition to any number of other parts that might affect functionality. A firm understanding of the interactions between the various components of a molecular device is essential before it can be fully realized and implemented. After being touted as possible candidates for molecular electronic devices for decades [1,2,3,4,5,6], spin crossover (SCO) molecules have shown potential recently for use in nonvolatile memory devices [7,8,9]. The SCO phenomenon is a transition between two spin states in a 3d transition metal ion coordination complex. The five, normally degenerate, 3d orbitals of the core metal split into three t_2g_ and two e_g_ like orbitals due to the ligand field [10,11,12] or due to an intramolecular electron transfer between a redox-active ligand and the transition metal center, which results in an internal charge redistribution corresponding to two different electronic isomers [13,14]. In these molecular compounds, depending on the ligand field strength and via an external stimulus, including light, temperature, electric field, and pressure, the transition metal ion can exhibit two different spin states dubbed the low spin (LS) state and the high spin (HS) state [15,16,17,18,19,20,21,22,23,24,25,26,27,28,29,30,31,32,33,34,35]. Such dynamic switching behavior of SCO molecular compounds [36,37,38,39,40,41] makes them candidates for molecular-based electronics such as sensors and fast optical devices [4,29,37,42,43,44,45,46,47,48,49,50], but maybe particularly suitable nanoscale transistors [44] and nonvolatile memory devices [7,8,9].

Although SCO complexes are promising candidates for use in various molecular-based devices due to their unique bistability properties, when these molecules are deposited as thin films onto a surface, parameters beyond the metal–ligand interaction arise which can drastically influence spin state switching and, in many cases, cause spin crossover complex to become locked in a given electronic state [42,51,52,53,54,55,56,57]. Thinking in terms of possible device applications, it is crucial to gain a better understanding of substrate influence. The interaction between molecular compounds and substrates has historically been among the most significant topics in material science as well as an abiding concern in device design [58,59,60,61]. It is impossible to make a device without interfaces as both electrodes and dielectrics are essential. Indeed, Nobel Laureate Herbert Kroemer coined the phrase “The interface is the device” [62]. For spin crossover complexes, the influence of a substrate can be highly variable. Substrates like graphite exhibit less influence on the spin state switching process of sublimated SCO thin films [26,57,63,64,65,66,67,68,69,70], while metallic substrates and some dielectric substrates can have a major effect on how an SCO molecule behaves [62,63,64,65,66,67,68,69,70,71,72,73,74,75,76,77], which has been revealed by different techniques including scanning tunneling microscopy (STM), X-ray absorption spectroscopy (XAS), X-ray magnetic circular dichroism (XMCD), X-ray photoelectron spectroscopy (XPS) [44,69,72,73,75,76,77,78] and so on. Additionally, oxide surfaces, due to their propensity for surface defects, may cause a lock in the sublimated SCO molecule in the specific spin state [5,77]. Some experimental and theoretical work suggest that a non-metallic coating layer, such as CuN and AlN, in between the SCO molecules and the metallic substrate reduces the interactions at the SCO-substrate interface [71,79,80]. Such a reduction in the influence of the subtrate may help adsorbed SCO molecules preserve their functionality.

While interaction of various SCO molecules with different surfaces has been discussed in prior reviews [5,54,63,64], a comprehensive review that focuses on the effect of different substrates on the functionality of SCO molecules does not yet exist. Here, the effect of different substrates on the spin states of different SCO molecules is reviewed more completely than in prior work. We detail both metallic and non-metallic substrates that lock the spin state, which is to say substrates that do freeze the spin state occupancy in one preferential combination of high and low spin states such that the spin state cannot be changed. Then, we provide counter examples of those substrates that do not lock the spin state. We also discuss ferroelectric substrate manipulation of spin states, to wit, the influence of the ferroelectric polarization direction of the spin state of an adjacent spin crossover molecular film. Moreover, the effect of film thickness, ligand modifications, thin film deposition method, surface morphology, and probing on substrate interactions is also discussed. Finally, and due to the fact that very little is well understood about the SCO-substrate interaction, we examine some insights provided by computational analysis and modeling. Obviously, with the increasing effort in spin crossover devices, these issues will have to be revisited if interest in spin crossover molecular thin film devices continues and understanding grows, but this review provides a start.

## 2. Metallic Substrates That Lock the Spin State

Metallic substrates tend to lock the spin state of SCO molecular compounds in a short range near the interface because of the strong coupling between SCO complexes and high electron density on metallic surfaces [5,81,82,83,84,85,86,87,88]. The coupling between a metallic substrate and an SCO molecule likely perturbs charges and the spin crossover molecular dipole. This substrate perturbation accordingly leads to the energy separation between LS and HS states. Therefore, it is difficult for an external stimulus to overcome the splitting energy and the molecule, becomes locked into one spin state. Some literature reports that Au(111) [5,78,82,83,84,85,86,87,88,89], Cu(100) [73,90], and Cu(111) [73] can cause a locking of the spin state of some SCO molecules. Of all the metallic substrates studied thus far, this locking effect occurs most prominently with Cu and Au substrates.

Cu is an example of a metallic substrate that typically locks the spin state of adsorbed SCO molecules, at least for the first molecular layer. [Fe(phen)_2_)(NCS)_2_] complexes were deposited on Cu(100), and the strong coupling between sulfur atoms of the NCS group to the Cu(100) substrate locked the spin state and prevented switching from HS to LS and vice versa [73,91]. On a layer-by-layer growth of [Fe(phen)_2_)(NCS)_2_] ultra-thin film on a Cu(100) substrate, spin-state coexistence was observed for the first monolayer and the HS/LS proportion was not correlated with molecular coverage. However, the second layer of [Fe(phen)_2_)(NCS)_2_] molecules experienced a weaker potential from the bottom blanket of phen orbitals produced by the first layer of Fe-phen which lead to a relaxation of any commensurability constraints on the adsorption geometries of [Fe(phen)_2_)(NCS)_2_]. The effect of different metallic substrates including Cu(100), Cu(111), Co/Cu(111), Co(100), Au(100), and Au(111) on spin state switching of [Fe(phen)_2_)(NCS)_2_] SCO molecular compounds revealed that the strength of the interactions between [Fe(phen)_2_)(NCS)_2_] and metallic substrates play a crucial role in SCO switching. Both LS and HS states could coexist, and fully switching between spin states was often not possible due to chemisorption [73].

**Figure 1:**
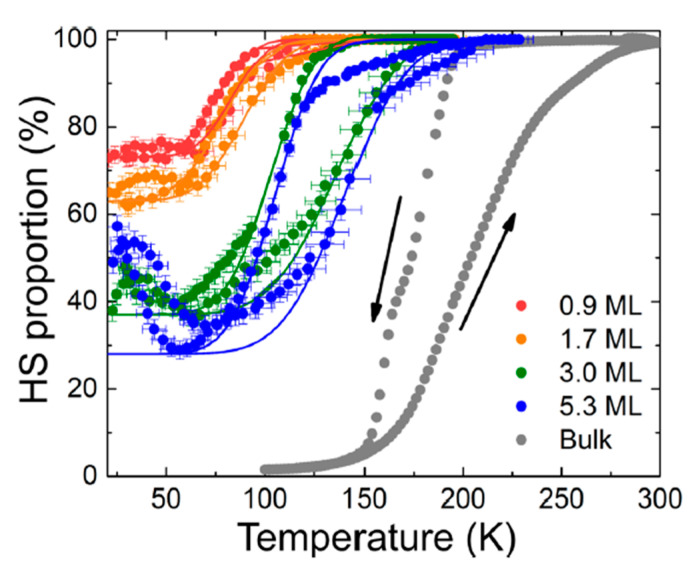
The HS percentage of Fe^II^((3,5-(CH_3_)_2_Pz)_3_BH)_2_ as a function of temperature; red, 0.9 ± 0.2 molecular layers; orange, 1.7 ± 0.4 molecular layers; green, 3.0 ± 0.7 molecular layers; blue, 5.3 ± 1.3 molecular layers (acquired from XAS spectra); gray, bulk form (taken from susceptibility measurement). Solid lines are for reference using an error function. Arrows indicate the direction of the changing temperature (increasing/decreasing temperature). Adapted with permission from reference [75].

Based on the Kelai et al. study, the transition temperature, the fraction of HS molecules at low temperature, and the bistability range of [Fe^II^((3,5-(CH_3_)_2_pz)_3_BH)_2_] SCO thin films on Cu(111) dramatically depends on the layer thickness (Figure 1) [75]. The thicker the deposited molecular layer, the lower the HS fraction and the higher the transition temperature. For temperatures lower than 50 K, the increase of the HS fraction is due to soft X-ray induced excited spin state trapping (SOXIESST) effects, though for thicker films this effect was found to be reduced significantly. The broadening of the hysteresis for several deposited layers of Fe^II^((3,5-(CH_3_)_2_Pz)_3_BH)_2_ revealed that the most striking is for a thermal hysteresis of 35 ± 9 K for a 3 molecular monolayer film which represents significant bistability for a thin SCO deposited layer of Fe^II^((3,5-(CH_3_)_2_Pz)_3_BH)_2_. Generally, as the molecular coverage increases, the temperature region for bistability occurs at larger temperatures, also indicating a persistent influence of the substrate.

**Figure 2:**
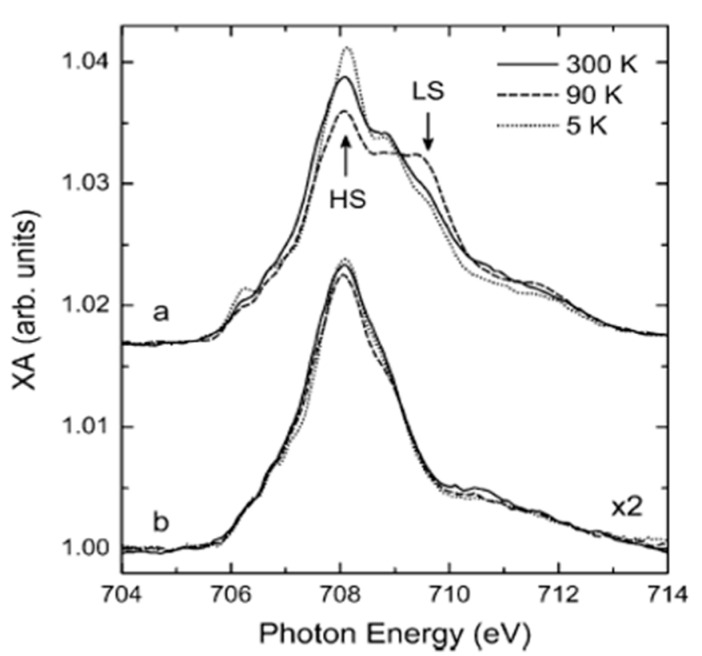
The Fe L_3_ edge NEXAFS spectra taken at 300 K, 90 K, and 5 K. (a) for 1.6 molecular layers and (b) for 0.8 molecular layers of [Fe(H_2_B(pz)_2_)_2_(phen)] on a Au substrate. Adapted with permission from reference [84].

A study by Gopakumar et al. [84], in which a few monolayers of [Fe(H_2_B(pz)_2_)_2_(phen)] (H_2_B(pz_2_) = dihydrobis (1-pyrazolyl)-borate), phen = 1,10-phenanthroline), deposited on an Au(111) substrate at room temperature, exhibited a different electronic structure from a bulk 6-coordinate complex where the ultra-thin film was not affected by temperature. Temperature-dependent near-edge X-ray absorption fine structure (NEXAFS) spectroscopy around the Fe L_3_ edge (Figure 2) showed that for 0.8 monolayers of [Fe(H_2_(bpz)_2_)_2_(phen)] on Au(111), the HS state dominated and the SCO molecular film did not undergo a spin state transition at low temperatures. However, for 1.6 monolayers, the LS peaks appeared in the X-ray absorption spectra at around 709.6 eV, which implies Au locks the molecules mostly in the HS state near the interface, while the molecules further from the surface are less affected and can undergo a spin state change with temperature.

Comparing the temperature-dependent XAS and XPS spectra of a monolayer ultra-thin film of [Fe(H_2_B(pz_2_)_2_(TTF)] (TTF = tetrathiafulvalene-fused dipyrido-[3,2-a:2′,3′-c] phenazine (dppz) ligand, on an Au surface with that of a thicker layer deposited on the same substrate, strong interactions at the interface to the Au was indicated [78]. The ultra-thin [Fe(H_2_B(pz_2_)_2_(TTF)] film loses the SCO functionality, with changing temperature, due to a strong interaction between the gold interface and sulfur atoms in the SCO ligand while the thick molecular film preserves switching between spin states. This indicates that metallic substrates tend to only lock the first few layers of the deposited SCO molecules in a short range near the interface.

In another study of [Fe(H_2_B(pz)_2_)_2_(phen)], a change in the current density of up to one order of magnitude and occurred over a very broad range of temperatures (100–300 K) with Au/[Fe(H_2_B(pz)_2_)_2_(phen)])]/GaIn junctions [92], in line with what would be expected for SCO complexes exhibiting a gradual spin state transition. The origin of this effect may be due to the strong coupling between the first few monolayers of sublimed [Fe(H_2_B(pz)_2_)_2_(phen)] thin film and the high electron density on the Au surface, leading to a spin state coexistence at a broad range of temperatures.

**Figure 3:**
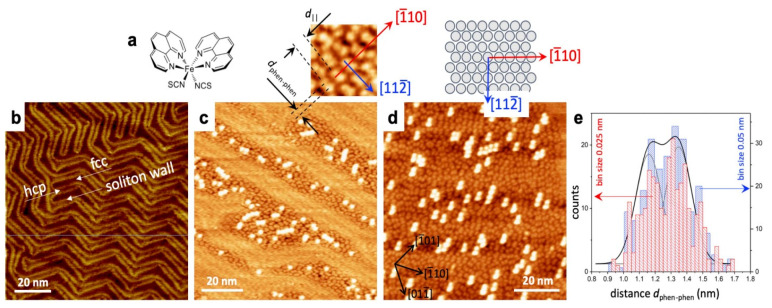
(**a**) A schematic structure of Fe (1,10-phenanthroline)_2_ (NCS)_2_([Fe(phen)_2_)(NCS)_2_]), referred in shortened form as Fe-phen. (**b**) An STM image of the Au(111) surface taken at 300 K. The ‘herringbone’ shape, typically associated with Au(111), is visible. (**c**) Approximately 0.3 molecular layers of Fe-phen on Au(111). The molecules look like two-lobe structures in the images. (**d**) Approximately full-monolayer coverage of Fe-phen. The above image: A magnified diagram of a sample area, with a similar structure model of an FCC (111) surface. Molecular rows are aligned along the (110) direction, with the molecules’ phen groups facing the (112) direction. (**e**) Histogram of d_phen–phen_ values, defined in (**a**) taken from multiple STM images. The histogram seems to indicate a binary distribution for d_phen–phen_, for two bin sizes, as shown. A double Gaussian peak is included for reference. STM scanning parameters: 600 pA, 1 V (**c**) and 500 pA, 1 V (**d**), and magnified area [85].

The spin states of [Fe(phen)_2_)(NCS)_2_] on Au(111) substrates in the sub-monolayer regime were mixed in a wide temperature range from 100 K to 300 K [85]. STM images show that sublimated [Fe(phen)_2_)(NCS)_2_] on Au(111) attaches to the Au through the NCS groups due to the strong affinity of sulfur binding to Au(111) which leads to the phen moieties pointing away from the Au surface. STM images of sub-monolayer [Fe(phen)_2_)(NCS)_2_] on Au (Figure 3a–d) showed that the [Fe(phen)_2_)(NCS)_2_] condenses into islands on the FCC sites of Au(111) with portions of the molecules in these regimes in the second layer just on top of the first layer islands. Compared to the Au surface in Figure 3b, the herringbone reconstruction is changed.

**Figure 4:**
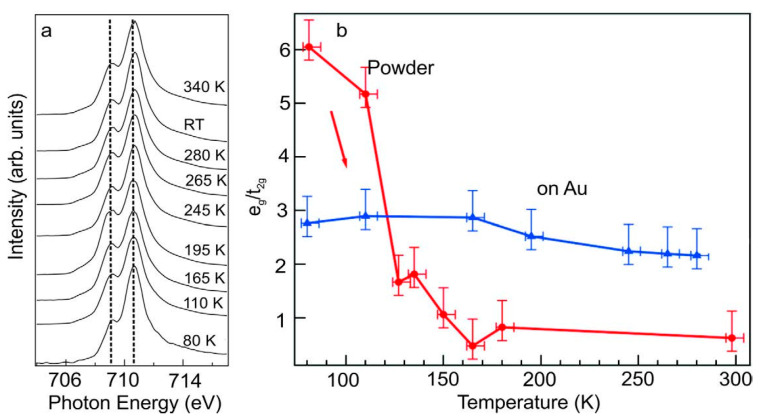
(**a**) The temperature dependent XAS of a bilayer [Fe(H_2_B(pz)_2_)_2_(bipy)] film on Au(111). (**b**) The temperature dependence of the unoccupied ‘e_g_/t_2g_’ state acquired from XAS for [Fe(H_2_B(pz)_2_)_2_(bipy)] powder (red circles) and molecular film on Au(111) (blue triangles). For both, the data was taken after reaching a temperature below 100 K, and then increasing the temperature to the indicated value. Adapted with permission from reference [88].

Ultraviolet photoelectron spectroscopy (UPS) measurements of [Fe(H_2_B(pz)_2_)_2_(bipy)] (bipy = 2,20–bipyridine) films, deposited on an Au(111), showed little evidence that the spin state occupancy changed at temperatures between 130 K and 300 K and this was attributed to the molecular packing effects that alter the cooperative effects compared to the bulk powder [86]. XAS indicated that while both the HS and LS states were represented, the low spin dominated over a temperature range beyond that associated with the spin transition of [Fe(H_2_B(pz)_2_)_2_(bipy)] molecules, when deposited on an Au(111) substrate [88]. XAS spectra of [Fe(H_2_B(pz)_2_)_2_(bipy)] thin films revealed that SCO molecules were in a mixed spin state at 340 K well above the typical transition temperature. After decreasing the temperature below the transition temperature, the spin state remained in a mixture of both HS and LS spin states (Figure 4a). The analysis of the XAS data for [Fe(H_2_B(pz)_2_)_2_(bipy)] revealed that the orbital occupation of e_g_ and t_2g_ is very sensitive to temperature. However, the occupation of the e_g_ and t_2g_ for [Fe(H_2_B(pz)_2_)_2_(bipy)], in a molecular bilayer film on Au(111), is independent of temperature as seen in Figure 4b. Here there is support for our contention that metals generally “lock” the spin state or mixed spin state and the spin crossover spin state change is suppressed.

Ossinger et al. [89] utilized two approaches in studying the reaction of the SCO complexes [Fe(H_2_B(pz)_2_)_2_(phenme_4_)] (pz = pyrazole, phenme_4_ = 3,4,7,8-tetramethyl-1,10-phenanthroline) and [Fe(H_2_B(pz)_2_)_2_(phen)] on Au(111) substrates. Evidence from both NEXAFS and STM shows that a sub-monolayer of this [Fe(H_2_B(pz)_2_)_2_(phenme_4_)] deposited on Au(111) dissociates into a four-coordinate complex, [Fe(H_2_B(pz)_2_)_2_], and phenme_4_. Similarly, the parent molecule [Fe(H_2_B(pz)_2_)_2_(phen)] dissociates into [Fe(H_2_B(pz)_2_)_2_] and phen on Au(111). For [Fe(H_2_B(pz)_2_)_2_(phenme_4_)] on Bi(111), a substantial fraction (approximately 50%) of the complex remains intact but remains locked in the high spin state independent of temperature [89]. In contrast with results on Au(111), temperature-dependent NEXAFS measurements of [Fe(H_2_B(pz)_2)2_(phenme_4_)] thin films on Bi(111) substrates show thermal, light, and X-ray-induced spin state switching.

Generally, metallic substrates lock at least the first few layers of SCO complexes into a specific or mixed spin state. However, there are a few reports of SCO molecules deposited on metallic substrates that can undergo spin state transitions. In such situations, other parameters beyond the SCO-metal interface interactions, such as high energy X-rays, the tips used for imaging, film thickness, and different types of ligands may be in play. In this section, we discuss how these parameters can affect the spin state transition of SCO molecules at temperatures ranging far from the transition temperature.

Although the conductivity of [Fe(H_2_B(pz)_2_)_2_(phen)] thin films sandwiched between indium tin oxide (ITO) and Al changes at different temperatures due to external light irradiation and this conductance change corresponds to a change in the spin state [93], this change in conductivity cannot confirm a complete switching to the other spin state or coexistence of HS/LS state. Furthermore, the transition temperature of bulk material is vastly different from that of thin film. While the bulk powder abruptly switches the spin state at 165 K, the thin film switches the spin state gradually somewhere between 100 K and 200 K, which could be due to the SCO-substrate interactions. Such a gradual switching of the spin state over a wide temperature range significantly increases the chance of HS/LS coexistence. Metallic substrates tend to only lock the first few adjacent layers of of a molecular SCO thin film at the interface. For the Fe(H_2_B(pz)_2_)_2_(phen)] thin films, sandwiched between indium tin oxide (ITO) and Al, the thin films were thick enough (10–100 nm) so at least some of the [Fe(H_2_B(pz)_2_)_2_(phen)] could switch spin state being located away from the interface [93]. It was claimed that ultra-thin films (5–6 molecular layers) of [Fe(H_2_B(pz)_2)_(phen)] deposited on Au(111) were determined to change from HS to LS during cooling, observed by UPS [94]. Interface effects can still be extensive. During cooling from 180 K to 60 K, an increase of the LS feature was observed, however, the transition temperature for the thin film was significantly lower than for bulk, implying an influence of substrate on the spin state [94]. In another work, Schleicher et al. [95] reported that a 42 nm thin film of [Fe(H_2_B(pz)_2_(phen)] sandwiched between two 20 nm Au electrodes showed evidence of a mixture of both HS and LS at room temperature.

The STM images of [Fe(H_2_B(pz)_2_(phen)] ultrathin films on Au(111) substrates show the change in the electronic structure of the second molecular layer that has been attributed to the transition between LS and HS states [96]. Coverages close to two monolayers of [Fe(H_2_bpz)_2_(phen)] were produced in an ultra-high vacuum environment by deposition on Au(111) surfaces at room temperature. STM images show that after cooling the sample to 5 K, the first monolayer orients with three pyrazole groups towards the substrate and the phenanthroline group away from the substrate. However, the second layer of [Fe(H_2_B(pz)_2_(phen)] molecules exhibits the opposite orientation. Sublimated monolayers of [Fe(HB(3,5-(CH_3_)_2_(pz)_3_)_2_] directly deposited on Au(111) revealed mixed spin states at low temperatures [74]. Although it was shown that a 50 nm [Fe(qnal)_2_] (qnal = quinoline-naphthaldehyde) thin film sublimated on an Au substrate behaves similarly to the bulk form [97], this observation could be due to the thickness of the film as the molecule locks mostly at the interface with the metallic substrate (as noted above). While based on temperature-dependent XPS and UPS measurements of a 6.7 nm [Fe(HB(trz)_3_)_2_] (HB(trz)_3_ = tris(1H-1,2,4-triazol-1-yl)borohydride) thin film, on Au it was claimed that the spin state switches from LS to HS [98], which confirms that Au substrates can mostly lock only the first few monolayers of the SCO molecules on and near the interface (less than 2 nm) and a minimum thickness of SCO molecules should be deposited to guarantee some SCO molecules were not locked into a specific spin state.

**Figure 5:**
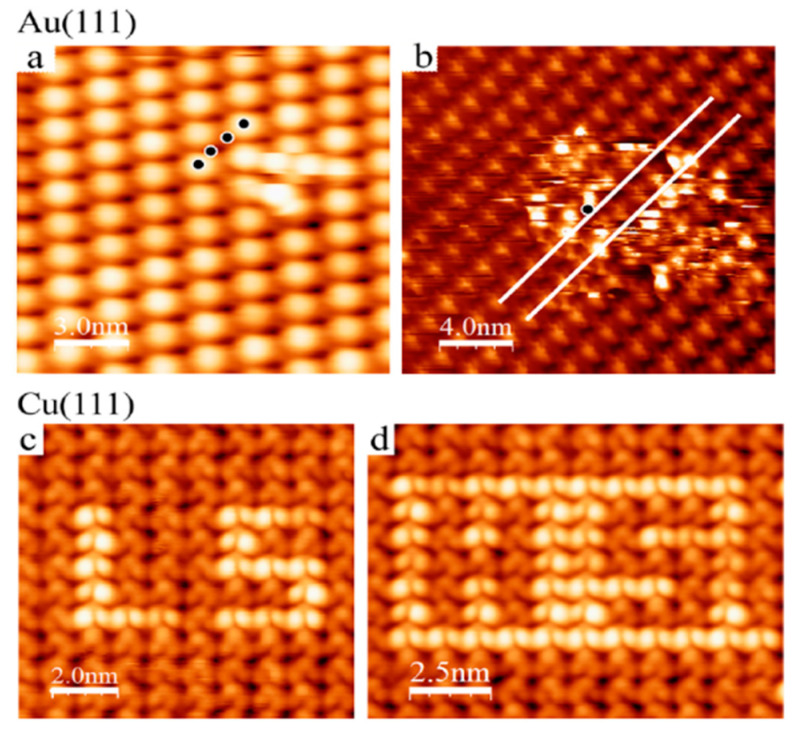
Voltage pulse induced switching of [Fe^II^((3,5(CH_3_)_2_pz)_3_BH)_2_] molecular layers (**a**) A 15 × 15 nm^2^ STM topograph acquired after four 0.6 V pulses applied at the points marked by black dots (V = 0.3 V, I = 20 pA, and z varies from 0 to 1.7 Å). (**b**) A 20 × 20 nm^2^ STM current image after a voltage pulse of 2.2 V (10 ms) applied at the point of the black dot (V = 0.3 V, ⟨I⟩ = 10 pA, and I vary from 0 to 250 pA). (**c**) 10 × 10 nm^2^ and (**d**) 10 × 12.5 nm^2^ topographs with “LS” and “HS” written by voltage pulses of 0.5 V (V = 0.3 V, I = 3 pA, and z varies from 0 to 2.63 Å). Adapted with permission from reference [99].

Voltage-induced STM spin state switching study of [Fe^II^((3,5(CH_3_)_2_Pz)_3_BH)_2_] on both Au(111) and Cu(111) was performed by Tong et al. [99]. On Au(111) surfaces it revealed that voltage pulses lead to the nonlocal switching of the molecules from HS to LS state or LS to HS state, even if it was possibly locked initially into a specific spin state due to strong coupling between SCO molecules and electrons on the metallic surface. However, on Cu(111) surfaces, [Fe^II^((3,5(CH_3_)_2_Pz)_3_BH)_2_] molecules maintained their electronic configuration after stimulation by a voltage pulse [99]. For the voltage pulses applied on molecules adsorbed on Au(111), a series of four consecutive pulses of 0.6 V (for 10 ms) was applied on four nearby molecules. The STM topographic images (Figure 5) show that the molecules on which the pulses have been applied are still in their initial state while a defect of bright molecules has appeared nearby, evidencing a nonlocal switching process. Voltage pulses lead to the switching of some molecules from the HS state to the LS state, however, single molecule bistability was not achieved within the 2D network on Au(111) because the original electronic state was recovered spontaneously (Figure 5a,b). On the contrary, the bistability of single molecules in the 2D network was noticed on Cu(111) surfaces. In Figure 5c,d, STM images reveal that all the molecules were switched to the HS state by a voltage pulse, then one by one, single molecules were switched back by voltage pulses to the LS state. This implies that not only is an external voltage needed to switch the spin state on metallic surfaces, but also there is a relatively stronger chemical interaction between [Fe^II^((3,5(CH_3_)_2_pz)_3_BH)_2_] and Au(111) substrates compared to Cu(111).

**Figure 6:**
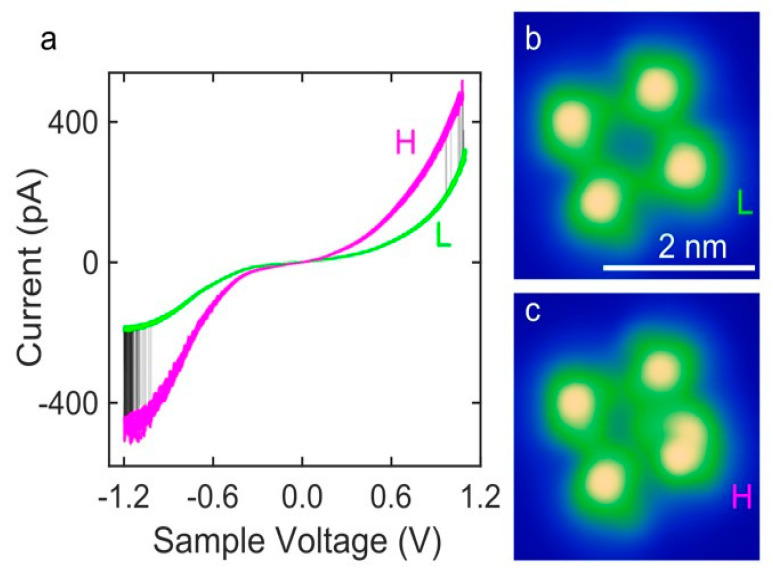
(**a**) The current as a function of voltage (I–V), taken for the spin crossover complex [Co(H_2_B(pz)(pypz))_2_], in a molecular tetramer, on an Ag(111) substrate. 16 different sweeps of the sample ramping between −1.2 to 1.1 V and back are shown. Quick transitions between the two states indicated as L and H are seen for |V| > 1 V, leading to a hysteresis. (**b**) An image of a tetramer before the application of a bias. (**c**) An image after applied bias (V = −1 V, I = −18 pA, duration 1 s) to the molecule denoted with an L in (**b**). In the H state, the molecule exhibits intramolecular contrast. Images were taken at −0.5 V and 10 pA. Adapted with permission from reference [100].

New functional Co-based SCO molecules, inspired by previous studies on Fe-based SCO molecules, opened another avenue for studying the effects of metallic substrates on the spin state transition. STM images of [Co(H_2_B(pz)(pypz))_2_] (py = pyridine, pz = pyrazole) molecular thin films deposited on Ag(111) substrates revealed that the molecules aggregate mostly into tetramers due to a bis (tridentate) coordination sphere [100]. [Co(H_2_B(pz)(pypz))_2_] tetramers on Ag(111) can exhibit a reversible spin transition between HS and LS states and can be stimulated by passing an external current through them or their neighbors. The current-voltage graph (Figure 6a) shows two spin states transitions that were measured repeatedly. While switching occurred at voltages greater than ±1 V, when the voltage was reduced, the spin state became locked (Figure 6b,c), indicating that a large external voltage stimulus is required for the spin state to change. Studies with [Fe(H_2_B-(pyrazole)(pyridylpyrazole))_2_] on an Ag(111) surface led to similar results [101,102].

## 3. Non-Metallic Substrates That Lock the Spin State

There is some evidence that oxides, including NiCo_2_O_4_, SiO_2_, Al_2_O_3,_ and LMSO (La_0.67_Sr_0.33_MnO_3_) [103,104,105] can lock the spin state of SCO molecules, due to surface defects that can lead to local charges or very strong interactions with the substrate and consequently may cause a locking of the SCO complex on the top layer. In the following, we review some of the molecules that were locked on such substrates.

**Figure 7:**
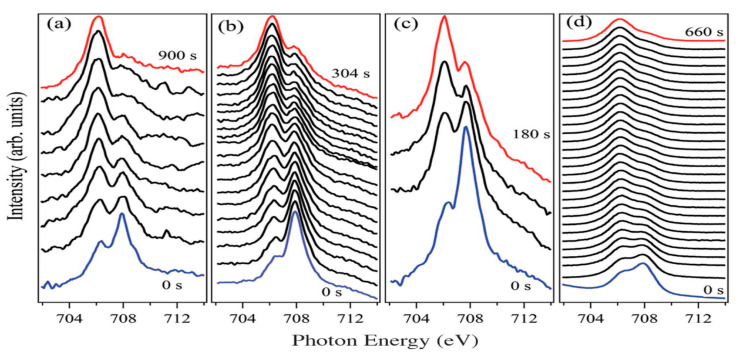
The XAS for thin films of [Fe(H_2_B(pz)_2_)_2_(bipy)] (**a**–**c**) on SiO_2_ and (**d**) on Al_2_O_3_ substrates, displaying time evolution of SOXIESST at (**a**) 200 K, (**b**) 290 K, and (**c**) 345 K for 5 nm films on SiO_2_. Comparable results are shown for (**d**) 30 nm films on Al_2_O_3_, at 294 K. From bottom to top, time increases, and the spin state changes from the LS state (blue) to the HS state (red). Adapted with permission from reference [77].

Locking of [Fe(H_2_B(pz)_2_)_2_(bipy)] thin films into the LS spin state was achieved by sublimating on dielectric substrates, such as Al_2_O_3_ and SiO_2_ [77]. Zhang et al. [77] reported that while XAS measurements of [Fe(H_2_B(pz)_2_)_2_(bipy)] powder show a successful spin state change with temperature, however, the 5 nm thin film of [Fe(H_2_B(pz)_2_)_2_(bipy)] sublimated on SiO_2_ does not display a thermally induced transition and remains unchanged from the low temperature up to room temperature, indicating that the film is mostly in LS at 290 K. Therefore, Si substrates can pin the spin state of [Fe(H_2_B(pz)_2_)_2_(bipy)] on the LS state for thin layers of 5 nm or less. The same experiment was done on 30 nm of [Fe(H_2_B(pz)_2_)_2_(bipy)] deposited on Al_2_O_3_. The spin state tended to be pinned in the LS state up to 345 K. Time evolution of the spectra during exposure of the film to X-rays during XAS at around 290 K shows a systematic behavior reminiscent of the unlocking of the spin state, allowing a transition to the HS state well above the transition temperature (Figure 7).

**Figure 8:**
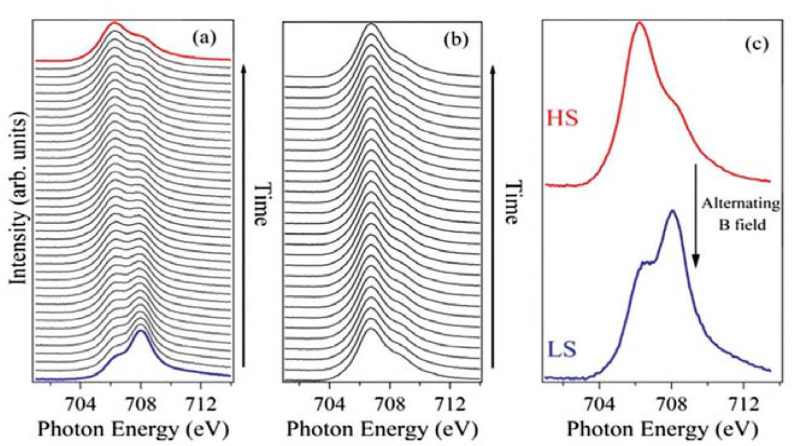
The time evolution of the XAS for a 10 nm thin film of [Fe(H_2_B(pz)_2_)_2_(bipy)] on LSMO. (**a**) The spin state changes from the LS state (blue) to the HS state (red) with time when exposed to X-rays and this occurs within 28 min. (**b**) De-excitation from HS to LS in an alternating magnetic field is not seen while subject to X-rays. (**c**) De-excitation occurs in the absence of X-rays in an alternating magnetic field. Adapted with permission from reference [103].

Magnetic oxide substrates have also exhibited a tendency to lock SCO complexes including [Fe{H_2_B(pz)_2_}_2_(bipy)] in the LS state [103]. A magnetic thin film of NiCo_2_O_4_ was deposited on Al_2_O_3_ followed by 10 nm of [Fe(H_2_B(pz)_2_)_2_(bipy)] via thermal evaporation. XAS data taken at a temperature above the thermal-induced transition temperature showed that the film was locked in the LS even at RT. With exposure to X-rays, the system switches from LS to HS and it can be switched back to LS by applying an alternating magnetic field. Furthermore, a [Fe(H_2_B(pz)_2_)_2_(bipy)] film sublimated on magnetic LMSO films on a SrTiO_3_ substrate exhibited the same behavior of locking the spin in the LS state (Figure 8).

Many experimental works show that graphite is an excellent substrate choice for preserving the functionality of SCO molecules, however, a few reports show graphite can affect the behavior of SCO molecular thin films [26]. While complete spin state switching was observed in bulk and in 10 nm thin films of [Fe(H_2_B(pz)_2_)_2_COOC_12_H_25_-bipy] on SiO_x_ wafers, in contrast, a spin-state coexistence of 42% LS and 58% HS was noticed for a 0.4 molecular layers deposition of the complex at 40 K on graphite. SOXIESST measurements revealed that cooling the sample to 10 K leads to an increase of the HS fraction to 64%, indicating the role of [Fe(H_2_B(pz)_2_)_2_COOC_12_H_25_-bipy] molecule-graphite interactions in tuning the thermal SCO characteristics of the complex. Computational studies show graphite as a 2D material can be a good substrate choice for SCO devices, however, it is still not entirely ideal for some SCO molecules as it causes the HS and LS splitting energy gap of specific SCO molecules to increase [104]. In addition, graphite is relatively expensive. We will discuss this in more detail later in this paper.

## 4. Substrates That Do Not Lock the Spin State

Some substrates including graphite, quartz, glass, CuN, tungsten diselenide (WSe_2_), and hafnium disulfide (HfS_2_) tend not lock adjacent SCO molecules is a specific spin state. A few of these are reviewed below. As mentioned earlier, there is some evidence that graphite might perturb the functionality of specific SCO molecules [104], however, like some other 2D materials graphene generally interacts less with sublimated SCO thin films [26,57,64,70,105] than most other substrates. Sub-monolayers of [Fe(NCS)_2_L] (L = 1-{6-[1,1-di(pyridin-2-yl)ethyl]-pyridin-2-yl}-N,N-dimethylmethanamine) deposited on graphite were switched repeatedly in a reversible manner between HS and LS by altering the temperature [65]. A carbon-based substrate in direct contact with the molecule preserves the SCO behavior.

**Figure 9:**
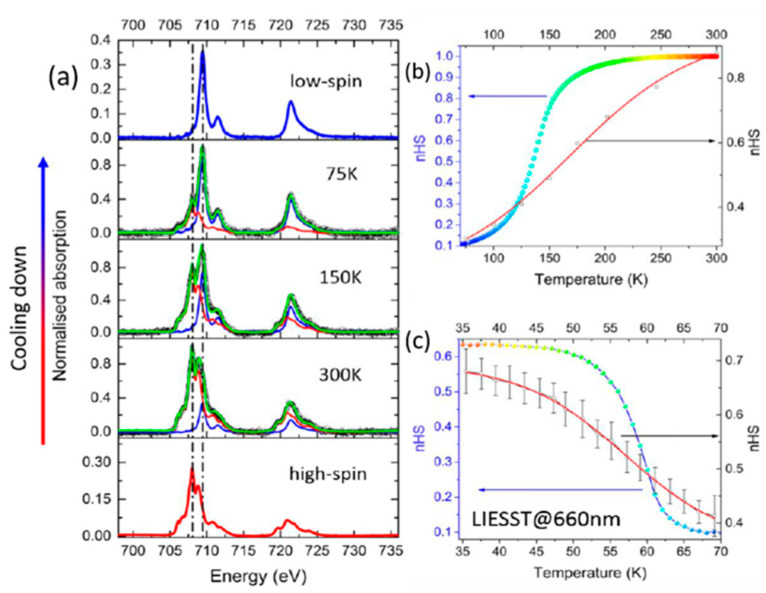
(**a**) The thermal evolution of the normalized Fe L_2,3_ edge XAS spectra for a single layer of [Fe(H_2_B(pz)_2_)_2_(phen)] (empty black dots) together with high-spin Fe(II) (red) and low-spin Fe (II) (blue) spectra taken from reference [15], used as reference signals for the spectral deconvolution (green lines). The broken lines are for reference. (**b**,**c**) The high-spin Fe(II) thermal distribution profile (empty circles) was obtained from XAS spectra taken before (**b**) and after (**c**) irradiation at 4 K by laser light. In (**b**,**c**) they are given by a Boltzmann distribution fitted line, giving a T_1/2_ = 168 + 15 K and T_1/2_ = 56 + 3 K, respectively. Gathered from reference for comparison is the data measured for the bulk sample plotted as colored dots. Adapted with permission from reference [107].

The spin state of [Fe(H_2_B(pz)_2_)_2_(phen)] sublimated on a graphite substrate can be switched via green light at 6 K and by increasing the temperature to 65 K [106]. A 0.7 sub-monolayer of [Fe(H_2_B(pz)_2_)_2_(phen)] sublimated on a graphite substrate can be switched at RT via light just like the bulk form and thick film [107]. Figure 9 demonstrates the temperature-dependence of absorption spectra at both the L_2_ and L_3_ edges of Fe of a submonolayer of [Fe(H_2_B(pz)_2_)_2_(phen)] on graphite, indicating successful switching between spin states. Recently [Fe(H_2_B(pz)_2_)_2_(phen)] was used in carbon nanotube nanoscale transistors [51] due to its functionality on carbon surfaces.

**Figure 10:**
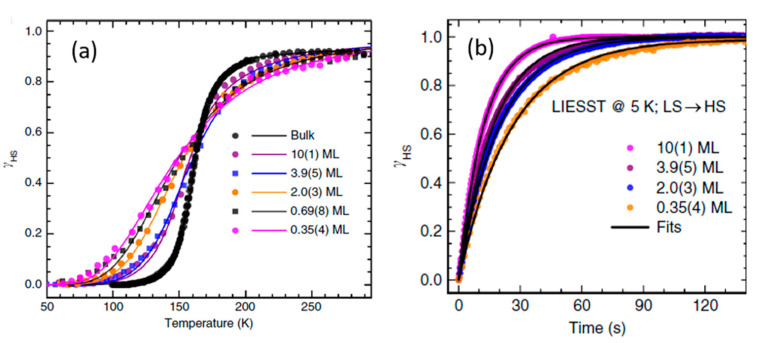
(**a**) The temperature-dependent spin crossover for different thicknesses of [Fe (bpz)-bipy] on highly oriented pyrolytic graphite compared to bulk; the dots are experimentally acquired data while the solid lines are fits that were derived from using the Slichter–Drickamer model. (**b**) The light-induced transition from LS to HS at 5 K for the different thicknesses [108].

The [Fe(H_2_B(pz)_2_)_2_(bipy)] molecule deposited on a graphite substrate successfully exhibited a complete thermal and light-induced spin transition at different thicknesses, with the width of the temperature-induced spin transition curve narrowing as the thickness was increased. The submonolayer exhibited a non-cooperative behavior, however, the multilayers exhibited a distinctly cooperative spin switching akin to free molecule behavior [108], including low-temperature light-induced excited spin state trapping (LIESST), that is to say, light induced excitation from the low spin state to the high spin state, an excited state where the molecule remains trapped at very low temperatures, as shown in Figure 10.

The magnetic moment of a [Fe(H_2_B(pz)_2_(phen)] thin film on a graphite substrate was changed via illumination by green light at 6 K [106], and by increasing the temperature up to 300 K. Around 90% of the SCO molecules were thermally induced, indicating that [Fe(Bpz)_2_phen] complexes preserve their spin switching when sublimated on a graphite substrate. Moreover, temperature-dependent XAS on [Fe^III^(qsal-I)_2_]NTf_2_] (qsal-I = 4-iodo-2-[(8-quinolylimino) methyl]phenolate) deposited on a layer of graphene confirmed that the [Fe^III^(qsal-I)_2_]NTf_2_] thin film switches from the HS to the LS state at lower temperatures when compared to the molecule in the bulk form [109].

**Figure 11:**
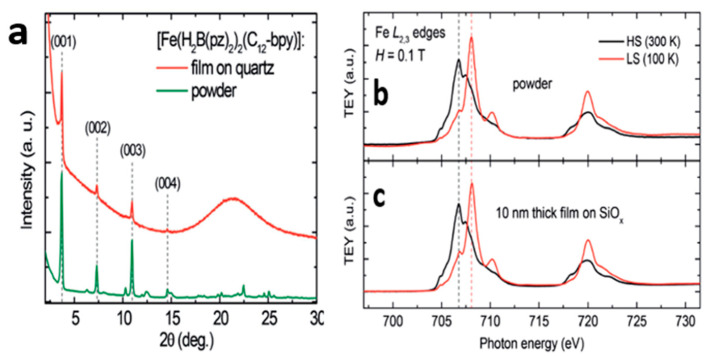
The self-assembly of a [Fe(H_2_B(pz)_2_)_2_(C_12_-bpy)] thin film. (**a**) The X-ray diffraction pattern of a 10 nm [Fe(H_2_B(pz)_2_)_2_(C_12_-bpy)] film on a quartz substrate with powder form for reference. The equidistant reflections (001–004) show the lamellar structure of 2.54 nm periodicity for both powder and film. The broad feature at 17–25° is evidence of the scattering signal from the quartz substrate. (**b**) The Powder and (**c**) thin film XAS at the Fe L_2,3_ edges of [Fe(H_2_B(pz)_2_)2(C_12_-bpy)]. The XAS spectra were obtained for powder (top) and 10 nm thick film on SiO_x_ (bottom) at 300 K and 100 K. The dotted lines indicate multiple features at ≈706.8 eV and ≈708.1 eV characteristics of HS and LS state molecules. The data was acquired in total electron yield mode at normal X-ray incidence and normalized to the sum of integrals over the Fe L_3_ and L_2_ edges. Adapted with permission from reference [110].

It was shown (Figure 11) that both temperature-dependent XAS and X-ray diffraction (XRD) spectra of [Fe(H_2_B(pz)_2_)_2_(C_12_-bpy)] thin films sublimated on glass substrates show the same SCO behavior as the powdered form [110], indicating that not only was the thin film sublimation a success (Figure 11a), but also the same behavior as the bulk molecule was witnessed (Figure 11b,c).

**Figure 12:**
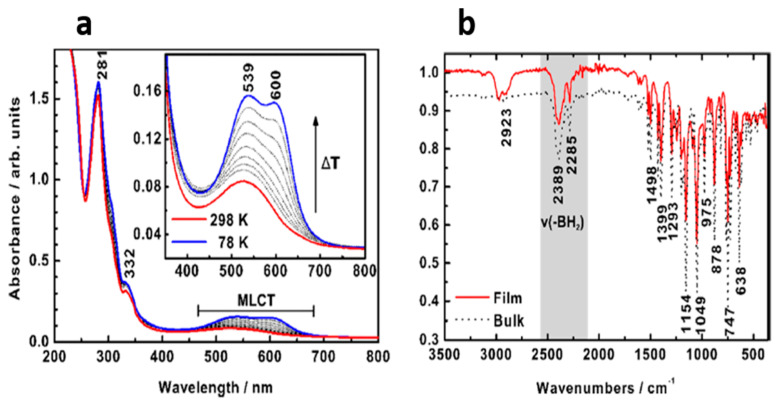
(**a**) The temperature-dependent UV-vis of a film of [Fe(H_2_B(pz)_2_)_2_(phenme_4_)] on a quartz disk at 298 K (red line) and 78 K (blue line). Gray: temperatures between 298 K and 78 K (**b**) The Fourier transform infrared (FT-IR) spectra of the bulk material (black dotted line) and the-deposited form (red line) of [Fe(H_2_B(pz)_2_)_2_(phenme_4_)] at 298 K. Adapted with permission from reference [89].

In another study, Naggert et al. [111] showed, by temperature-dependent UV-Vis spectroscopy, that a 480 nm film of [Fe^II^((H_2_Bpz)_2_)_2_(phen)] deposited on glass can undergo a change in spin state with changing temperature. [Fe((H_2_B(pz)_2_)_2_(bipy)] evaporated on glass behaved similarly. These results show that glass can be a proper candidate for molecular-based devices. Just like its parent molecule ([Fe(H_2_Bpz)_2_)_2_(phen)]) discussed above, spin state switching of [Fe(H_2_B(pz)_2_)_2_(phenme_4_)] deposited on quartz was seen to change spin state in temperature-dependent UV-Vis spectroscopy (Figure 12a) [89]. The greater crystallinity of quartz, compared to glass, may influence the effective cooperativity in the SCO film adjacent to the interface [112]. Additionally, the Fourier transform infrared (FTIR) spectra of [Fe(H_2_B(pz)_2_)_2_(phenme_4_)] are very similar to the bulk form, indicating that the molecule maintains its crystalline structure after sublimation (Figure 12b).

**Figure 13:**
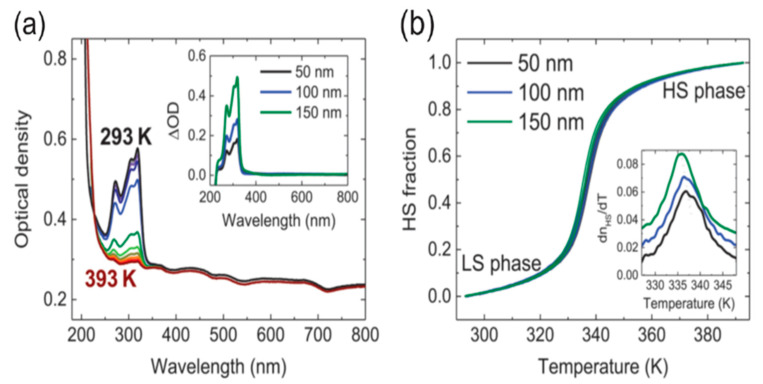
(**a**) The optical absorbance spectra of the 100 nm [Fe(HB(trz)_3_)_2_] film were obtained at specific temperatures between 293 K (LS) and 393 K (HS). Inset: optical density changes ΔOD = OD_293K_ − OD_393K_ of all three films as a function of wavelength. (**b**) The temperature dependence of the HS fraction for all three films was acquired by varying the optical density at λ = 317 nm during one heating-cooling cycle at a rate of 1 K min^−1^. The inset displays the derivatives of the transition curves. Adapted with permission from reference [113].

[Fe(HB(trz)_3_)_2_] spin crossover thin films deposited on silica substrates, with thicknesses, 50 nm, 100 nm, and 150 nm, were successfully switched between the two spin states by altering the temperature [113]. While the transition temperature is well above RT (375 K) and initially the molecule is in LS, optical absorbance spectra revealed that the molecule was switched to HS by increasing the temperature. This behavior indicates that switching is independent of film thickness (Figure 13).

**Figure 14:**
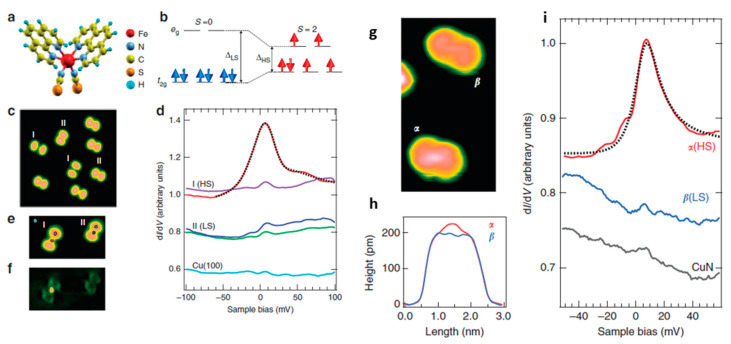
[Fe(phen)_2_)(NCS)_2_] SCO molecules deposited on Cu(100). (**a**) A 3-D image of this Fe-phen molecule. (**b**) The LS and HS electronic configurations of the FeII 3d orbitals. (**c**) An STM image of Fe-phen molecules on Cu(100) with two forms denoted as I (HS) and II (LS). Image dimensions are 13 × 13 nm^2^. (**d**) The dI/dV spectra taken at the center of type I (HS), type II (LS) molecules, and the Cu(100) surface. The colors indicate the points where the spectra were acquired and are marked as colored dots of (**e**). The black dotted line represents a Fano fit to the Kondo resonance. (**e**) An STM image of a pair of type I (HS) and type II (LS) molecules and their (**f**) related dI/dV map (bottom) obtained at +10 mV indicating the point of the Kondo resonance at the center of the HS molecule. Image dimensions are 6.7 × 3.7 nm^2^. Fe-phen SCO molecules deposited on CuN/Cu(100). (**g**) An STM image (3 × 5.5 nm^2^) of single Fe-phen molecules on the CuN/Cu(100) surface with two forms listed as α (HS) and β (LS) and (**h**) line scans across the long axis of the molecules indicating their difference. (**i**) The dI/dV spectra taken at the center of the two configurations of the Fe-phen molecules together with a Fano fit (dotted black line) to the Kondo resonance of type α. The black line is the spectrum recorded on bare CuN. Adapted with permission from reference [71].

A study done by Miyamachi et al. revealed that surface interactions can be affected by having an interfacial layer. A film of [Fe(phen)_2_)(NCS)_2_] deposited on a thin interfacial layer of CuN on a Cu(100) surface significantly decreases the interaction between surface and molecule and allows the [Fe(phen)_2_)(NCS)_2_] to switch from HS to LS and vice versa due to a reduction of the adsorption energy [71]. By using STM, the stimulus was focused on the area of current flow which demonstrates electronic switching of the spin state. While Fe-phen molecules sublimated onto a Cu(100) surface showed mixed HS and LS states due to the strong coupling of the NCS group to the substrate, it prevented electronic switching of the spin state. However, a thin CuN layer deposited on Cu(100) allowed switching between the HS and the LS state. A monoatomic CuN layer dramatically reduces the adsorption energy and ensues hybridization, reducing the chemical interaction between molecule and substrate and weakening the bond of sulfur to the oxidized Cu atoms in the CuN network. As with Cu(100), STM images (Figure 14), revealed that [Fe(phen)_2_)(NCS)_2_] adsorbs with the NCS groups onto the CuN surface, and two types of molecular conformations (α and β) were observed. Due to the electronic decoupling of the molecules, the difference in the molecular shape seen by STM was dramatically smaller when compared with that on bare Cu. Figure 14h shows two slightly different height profiles in the center region, with type α higher than that of type β. Figure 14i represents the dI/dV spectra near the Fermi energy on the center of both types demonstrating a clear Kondo resonance only on type α and also a weak spectroscopic property on type β. Except for CuN, similar studies show sublimated SCO complexes on the family surface group like Cu_2_N preserve their functionality [114].

Substrates possessing a lower density of states near the Fermi level can decrease the van der Waals interaction at the SCO/substrate interface and can preserve the functionality of the deposited SCO thin films [57,89]. Previously, we saw [Fe(H_2_B(pz)_2_)_2_(phen)] deposited on Au(111) became pinned in a given spin state, however, a partial spin state switching was noticed for the same molecule deposited on a Bi(111) substrate [89] which is a semimetal with a relatively lower density of states than Au(111) at the Fermi level [89]. Investigating this further, Rohlf et al. [57] deposited [Fe(pypyr(CF_3_)_2_)_2_(phen)] (pypyr = 2-(2′-pyridyl) pyrrolidine) on the semiconducting layered dichalcogenide materials WSe_2_ and HfS_2_, both of which have a reduced density of states at the Fermi level, and witnessed a full spin state transition. Then, they compared the HS fraction of [Fe(pypyr(CF_3_)_2_)_2_(phen)] deposited on Au(111) and graphite, respectively. XAS measurements demonstrated that the spin state of the [Fe(pypyr(CF_3_)_2_)_2_(phen)] thin film deposited on Au(111) was locked. However, a successful transition to the HS state was noticed for [Fe(pypyr(CF_3_)_2_)_2_(phen)] deposited on graphite, WSe_2,_ and HfS_2_ confirming the previous results [57].

A change in the spin state of an SCO thin film usually causes a noticeable change in the electrical conductivity and charge transport properties as well [48]. Generally, glass, between interdigitated Au electrodes, seems to be a promising substrate as thin films on such substrates tends to not affect the deposited molecule’s ability to switch spin states. The conductivity of [Fe(HB(pz)_3_)_2_] and [Fe(H_2_B(pz)_2_)_2_(bipy)] thin films on such substrates with gold interdigitated microelectrodes will change spin state occupancy when the temperature is altered [8,115,116]. For the substrates with interdigitated gold electrodes, the substrate is only partially occupied with the gold microelectrodes and most SCO molecular thin film is not in direct contact with the gold. Computational studies show SCO molecules between interdigitated microelectrodes may not act independently and can couple with each other, leading to a change in their properties [117]. This will be further discussed below (vide infra).

Switching of the spin state of [Fe(Htrz)_2_(trz)](BF_4_)] by an induced electric field on Si/SiO_2_ substrates with interdigitated Au microelectrodes, with an inter-electrode gap of 4 micrometers, has been reported in Lefter et al. [118]. Transmission electron microscope (TEM) measurements of [Fe(Htrz)_2_(trz)](BF_4_)] molecules revealed that the cooperative SCO behavior of such nanocrystals is altered by changing temperature, which confirms that the breathing of the crystallographic unit cell shows up at the nanoscale [119]. Beyond that, probing this length change, the photo-switching dynamics of a single nanoparticle and how it was affected by the presence of gold nano-rods using an ultrafast transmission electron microscope (UTEM) revealed that increasing the number of gold nano-rods accelerates the photo-switching rate.

[Fe(pyrazine)Pt(CN)_4_] thin films created by laser-mediated evaporation on Si substrates showed an altered SCO behavior going from a sharp transition with temperature hysteresis of 16 K around room temperature in the nanocrystalline form to a gradual transition shifted downwards to 170 K in thin film form due to a side effect of laser-induced desorption. However, XRD and microscopic imaging showed both the nanocrystalline and thin film forms have similar structures [120]. The light-induced spin transition mechanism, due to the coupling between [Fe[HB(3,5-(Me)_2_Pz)3]_2_] SCO complexes and 2D materials, allows the efficient optoelectrical detection of the spin transition. It was shown that the spin state of the [Fe[HB(3,5-(Me)_2_Pz)_3_]_2_] complex in the SCO-graphene substrate interfaces changes due to electrical detection at the interface [121].

The angle-resolved X-ray photoelectron spectroscopy (ARXPS) measurements of [Fe^III^(qsal-I)_2_]NTf_2_] SCO complex on a Cu/SLG/[Fe^III^(qsal-I)_2_]NTf_2_]/GaOx/EGaIn SCO molecular junction device show that the counteranion was adsorbed on the graphene with the [Fe(qsal-I)_2_] plus cation on top, which caused the SCO molecule to decouple from the Cu electrode. XAS and XMCD spectroscopy showed reversible spin state transition in the molecular junctions. Gakiya-Teruya et al. [122] prepared thin films of the [Fe(tBu_2_qsal)_2_] SCO complex at a sublimation temperature of 423 K and background pressure of 10^−8^ mbar onto a Pt/Ti/SiO_2_/Si(100) substrate. Following frequency-dependent capacitance measurements and a hysteretic spin transition, they concluded that the Pt layer did not affect spin state switching.

Poggini et al. [98] reported on a 100 nm film of [Fe(HB(tz)_3_)_2_] thermally evaporated on a Kapton substrate, the spin crossover transition is incomplete at 350 K, different from the switching of bulk [Fe(HB(tz)_3_)_2_] with a transition temperature of 326 K. This change and gradual spin state transition in the film compared with the bulk material can be due to the effect of nano-structuration on the compound or even may arise from a relevant increase of surface energies when going from LS to HS. A successful temperature-dependent charge transport measurement of a [Fe(HB(tz)_3_)_2_] thin film evaporated on 180 nm lithography patterned ITO electrodes on a glass substrate was achieved [123]. For the different thicknesses of [Fe(HB(tz)_3_)_2_], thin film spin transition at different temperatures occurred, however for the film with 10 nm thickness no spin transition was detected.

## 5. Ferroelectric Substrate Manipulation of the Spin State

**Figure 15:**
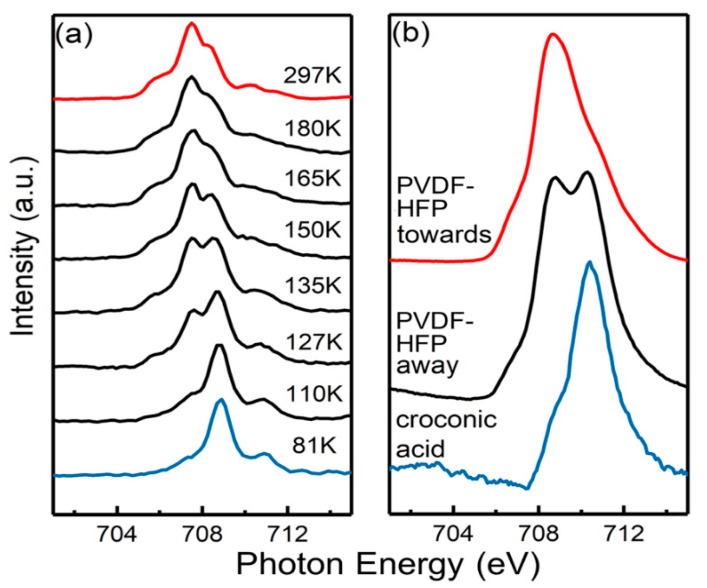
The temperature-driven SCO transition (**a**) compared to the electric field control of the spin state of [Fe(H_2_B(pz)_2_)_2_(bipy)] thin films on PVDF-HFP ferroelectric substrates (**b**). The XAS reveals that [Fe(H_2_B(pz)_2_)_2_(bipy)] is pinned for the most part in the LS state when the ferroelectric polarization of PVDF-HFP has pointed away from [Fe(H_2_B(pz)_2_)_2_(bipy)] (PVDF-HFP away) and converts to the HS state when the ferroelectric polarization of PVDF-HFP is pointing towards [Fe(H_2_B(pz)_2_)_2_(bipy)] (PVDF-HFP towards). [Fe(H_2_B(pz)_2_)_2_(bipy)] on croconic acid persists in the LS state, in the absence of an applied voltage (croconic acid). Adapted with permission from reference [9].

While several methods have been suggested to control switching between spin states [112,124], it has been found that by changing the ferroelectric polarization, the spin state occupancy of a very thin spin crossover molecular film can be manipulated [9,125,126,127]. Thus, one possibility is using a ferroelectric material in the interface [127,128]. Ferroelectric substrates are an option to control the spin state of a particular SCO thin film layer. In other words, the influence of the substrate, favoring one particular spin state, depends on the ferroelectric polarization. The organic ferroelectrics poly(vinylidene fluoride-hexafluoropropylene) (PVDF-HFP) and croconic acid can both lock the spin state of the spin crossover complex [Fe(H_2_B(pz)_2_)_2_(bipy)] thin films depending on the ferroelectric layer polarity (Figure 15a,b) [9].

The thickness of the PVDF-HFP layer in PVDF-HFP/[Fe(H_2_B(pz)_2_)_2_(bipy)] bilayer films plays a crucial role in achieving an optimal functionality of switching to different spin states by altering the polarity [125]. Moreover, the spin state of the [Fe(H_2_B(pz)_2_)_2_(bipy)] thin film tends to be locked in a specific spin state depending on the direction of the polarization at the interface with organic ferroelectric PVDF-TrFE (polyvinylidene fluoride with trifluoroethylene) [126].

**Figure 16:**
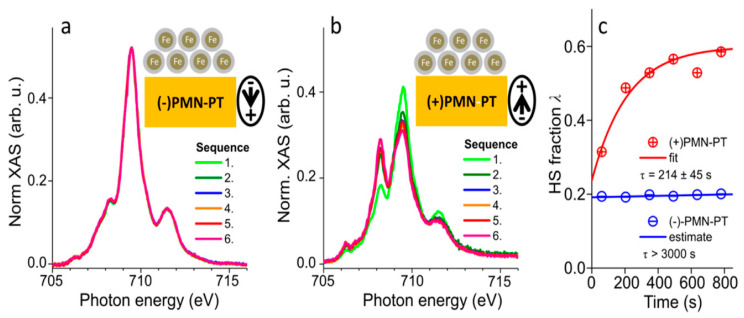
The XAS spectra for [Fe(H_2_B(pz)_2_)_2_(bipy)] spin crossover molecular thin films at 3 K on the ferroelectric oxide (+)- and (-)-PMN-PT. (**a**,**b**) A progression of six different spectra on both substrates showing the Fe L_3_ edge. At ϕ0 = 0.14 ph s^−1^ nm^−2^, each spectrum corresponds to an X-ray quantity of D = 20 ph nm^−2^. Spin-state trapping is seen on (+)-PMN-PT, but not on (−)-PMN-PT. The spectra are normalized for clarity. (**c**) Time-dependent HS fractions were gathered from the spectra in (**a**,**b**). Adapted with permission from reference [127].

[Fe(H_2_B(pz)_2_)_2_(bipy)] complexes deposited on differently poled ferroelectric PMN-PT ([Pb(Mg_1/3_Nb_2/3_)O_3_]_1−x_[PbTiO_3_]_x_, x = 0.32) characterized by X-ray absorption spectroscopy revealed temperature-driven conversion between HS and LS states with no observable effect of the ferroelectric polarization on the spin state of the molecules down to 100 K [127]. However, at 3 K, large differences were noticed between the two ferroelectric polarizations. At this temperature, the efficiency of X-rays exciting the molecules to the HS state was more than an order of magnitude larger when the ferroelectric dipoles of the substrate were pointing toward the surface, compared to the opposite polarization. SOXIESST in the thin film samples at 3 K exhibited no SOXIESST on the (−) poled substrate, whereas on the (+) poled sample, a significant HS fraction appears within minutes of irradiation. Figure 16a,b display two sequences of six X-ray absorption spectra each on negative and positive PMN-PT. These results were confirmed in the HS fractions overtime plot (Figure 16c). Surprisingly, the high spin state of [Fe(H_2_B(pz)_2_)_2_(bipy)] favors the ferroelectric being poled “up” and the low spin state favors the oxide ferroelectric being poled down [127], which was also seen for [Fe(H_2_B(pz)_2_)_2_(bipy)] on the organic ferroelectrics PVDF-HFP and PVDF-TrFE [7,8,9,126].

1500 nm thick [Fe(Htrz)_2_(trz)](BF_4_)] thin films on PVDF-TrFE polymer were fabricated as bilayer samples to be used potentially in MEMS devices [128]. A reproducible actuation near the transition temperature was seen in the composite which proved the conservation of the thermal actuating characteristics of the nanoparticles in the thin film, which means PVDF-TrFE can influence SCO samples with thicknesses greater than a micron [128], although this may in effect be a pressure effect, suggesting that magneto-striction could be very real in such heterolayer devices.

## 6. Other Parameters That Influence the Spin State Switching

In the simplest picture, it would be expected that the influence of the substrate extends only to the layer of SCO molecules in direct contact with the substrate, but in reality, the situation is far more complicated. To better understand how different substrates can affect the functionality of SCO complexes, careful attention to other parameters that can potentially perturb SCO molecules should be considered. These include ligand types, film thickness, thin film fabrication method, surface properties of the substrates, and the probing methods used [91,129,130,131,132,133,134,135,136,137,138].

Doping SCO complexes with certain chemicals can alter properties such as transition temperature and conductivity [112,139,140]. Fluorination of specific SCO complexes can improve their functionality in thin film form [91]. Polymorphism can drastically influence the crystal packing structure and the physical properties of SCO molecules. A change in packing mode can lead to a change in intermolecular interactions, and eventually cause a critical shift in transition temperature [40,129,130,131]. Recently, chemists have tried to synthesize new SCO complexes for use in spintronic devices which are compatible with metallic surfaces such as Au and Ag [141]. Minor ligand modifications in Fe (II) SCO complexes [Fe(H_2_B(pz)_2_)_2_(L)] can lead to significant changes in the SCO properties of the molecules. Temperature-dependent UV-Vis spectroscopy on both bulk and thin films revealed that the transition temperatures of these two phases were different [68], which is similarly true for other molecules [142].

**Figure 17:**
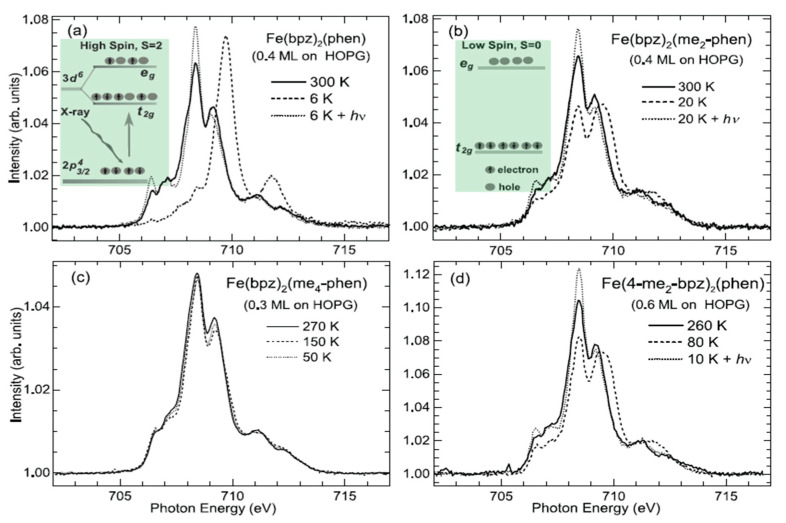
The XAS L_3_-edge spectra of: (**a**) 0.4 molecular layers of [Fe(bpz)_2_(phen)] deposited on graphite; (**b**) 0.4 molecular layers of [Fe(bpz)_2_(Me_2_-phen)] on graphite; (**c**) 0.3 molecular layers of [Fe(bpz)_2_(me4-phen)] on graphite, and (**d**) 0.6 molecular layers of [Fe(4-Me_2_-bpz)_2_(phen)] on graphite. The inset of (**a**) illustrates the high-spin configuration with electrons occupying both the e_g_ and t_2g_ levels, while the inset of (**b**) illustrates the low-spin configuration with the lower t_2g_ level fully occupied by electrons. Adapted with permission from reference [66].

Beyond regular spin state switching methods in SCO molecules, it is also possible to switch the spin state by coordination/decoordination of ligands in the molecule [96]. This has been shown to influence the cooperativity of the deposited SCO complexes [132,143]. Thermal and light-induced [Fe(H_2_B(bpz)_2_)_2_(phen)] complexes and their methylated derivatives on graphite substrates [66] show that while the unmodified complexes potentially show both thermal and light-induced spin state transition, the addition of a few methyl groups leads to a loss of the SCO on the surface (Figure 17a–d). Moreover, comparing angle-dependent measurements of K-edge with calculations leads to the conclusion that both switchable SCO molecules and those molecules locked in their HS state on the surface have a similar preferential orientation, but molecules with an incomplete SCO show a random orientation on the surface.

The method of thin film deposition can significantly affect the functionality of SCO complexes [133,134,135,136,137]. An uncontrolled SCO film thickness results in dramatic changes in thickness and cooperativity between molecules and consequently, the SCO properties may be altered. Precise control of the thickness is crucial as changes in the thickness can influence the functionality of the deposited SCO complex, especially for fabricating 2D SCO film complexes [144,145,146,147].

Minimum cooperativity between SCO molecular thin films is desirable for fabricating nano sized sensors [148]. An incomplete spin state switching was reported for bulk [Fe(H_2_B(4-CH_3_-pz)_2_)_2_(bipy)] due to intermolecular interactions, however, for vacuum-deposited thin films a complete switching was seen due to a decrease in intermolecular interactions [149]. The film thickness of the [Fe(H_2_B(pz)_2_)_2_(bipy)] molecule plays a crucial role in the tunability of the energy barrier between states due to the interactions at the [Fe(H_2_B(pz)_2_)_2_(bipy)]/Al_2_O_3_ interface. The bistability of the spin state hysteresis for films with thicknesses (300 nm and 900 nm) implies that the temperature range of the bistability can be broadened for different film thicknesses [132].

Layer by layer deposition is one of the methods used to fabricate SCO thin films with highly controlled thicknesses in the range of a nanometer [150]. This method was utilized for fabricating heterostructured SCO thin films that contained a well-defined buffer layer between the metallic substrates and SCO molecules. The result was a dramatic change in the SCO transition temperature [136]. The film thickness of crystalline ultrathin films of the SCO [Fe(py)_2_[Pt(CN)_4_] fabricated by the layer-by-layer method significantly affects the spin transition of the molecule [151]. For ultra-thin films below 10 nm, the functionality of the SCO complex is harshly affected, however for samples with thicknesses higher than 10 nm a similar behavior as a bulk complex is noticed. This can be due to an enhancement in cooperativity between molecules as a result of interparticle interactions. However, for films below 10 nm, SCO molecules become locked in the HS state as crystallites surrounded by Fe centers are partially separated [149,151]. STM images that compared to the deposition by sublimation revealed that the electrospray ionization deposition thin film preparation method of [Fe(pap)_2_]^+^ results in a significantly higher proportion of intact molecules on the Au(111) surface [77].

[Fe^II^(Htrz)_2_(trz)](BF_4_)] molecules spray-coated on SU-8 (an epoxy-based photoresist designed for micromachining) polymer surfaces in micrometer scale thicknesses result in smooth and homogenous films that can be used in both microscopic and macroscopic actuator devices. The thermal hysteresis loop of the composite film with twice as large as comparing the initial nanoparticles was achieved due to the combined effects of thickness mechanical interactions in the interface of [Fe^II^(Htrz)_2_(trz)](BF_4_)]/SU-8 [152]. The transition temperature of [Fe(pypyr(CF_3_)_2_)_2_(phen)] drastically decreases by around 60 K in thin film form on a substrate, compared with the bulk form [72]. Moreover, thin films of [Fe(pypyr(CF_3_)_2_)_2_(phen)] showed sensitivity to the SOXIESST effect in the temperature range of 28 K to 90 K with switching from LS to HS state and vice versa drastically improved due to reducing the internal pressure of the molecule in thin film form.

**Figure 18:**
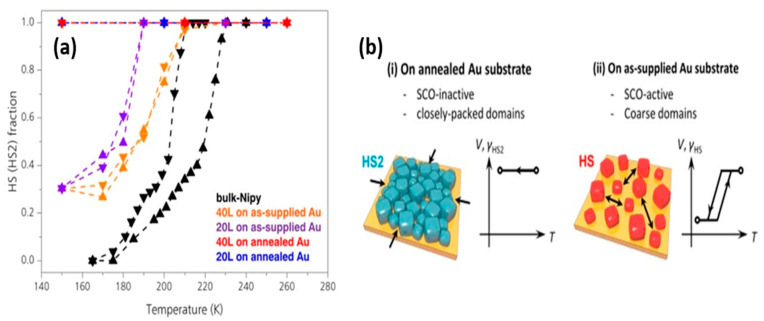
Spin crossover behavior for ultrathin [Fe(py)_2_[Ni(CN)_4_]] films. (**a**) The spin transition curves as a function of temperature for bulk-Ni py and film-Ni py on unannealed/annealed Au substrates. Triangles and inverted triangles represent heating and cooling processes. (**b**) The surface structure guided spin state of films in (i) SCO-inactive HS and (ii) HS states. Adapted with permission from reference [138].

The effect of surface morphology on the SCO complex [Fe(py)_2_[Ni(CN)_4_]] was established by comparing the SCO behavior when on annealed and unannealed (as-supplied) Au substrates [138]. The spin transition curves of [Fe(py)_2_[Ni(CN)_4_]] are shown in Figure 18a,b for thin films on as-supplied substrates and the transition temperature shifted to lower temperatures with the more imperfect substrate crystallite domain size decreased. On the other hand, 40-layer and 20-layer thin films deposited on annealed Au substrates showed no SCO phenomena when changing the temperature.

**Figure 19:**
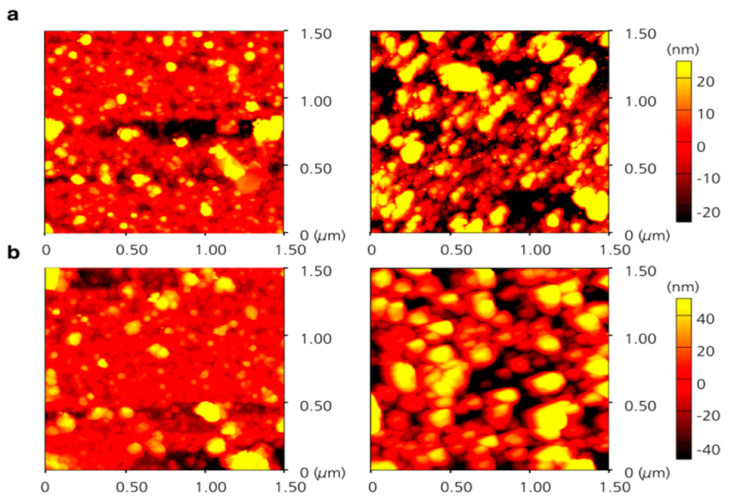
The surface morphology of the deposited films. Topographic AFM images were acquired in tapping mode for (**a**) 20 layers of film-Nipy and (**b**) 40 layers of film-Nipy which were grown on a SAM-anchored Au(111) surface. Images on the left correspond to untreated Au substrates. On the right, substrates were H_2_ annealed. Adapted with permission from reference [138].

This phenomenon can be explained by AFM studies (Figure 19a,b). Crystal domains of {Fe(py)_2_[Ni(CN)_4_]} on annealed Au substrates mostly aggregated in comparison with coarse granular-like particles when on the as-supplied Au substrate, which leads to the conclusion that the surface microstructure can affect the stabilization spin state, in thin film form [138]. This lends further support to the fact that Au, as a substrate can lock the spin state, as discussed above.

Sub-monolayers of [Fe(HB(3,5-(CH_3_)_2_(pz)_3_)_2_] on the metallic substrates Au(111), Ag(111), and Cu(111) have also been investigated. Zhang et al. [153] proposed a new mechanism, for the spin state transition of [Fe(HB(3,5-(CH_3_)_2_(pz)_3_)_2_], based on light absorption by the substrate that generates vibrational excitations which in turn may lead to spin state switching in the SCO-metallic substrate interface. They applied both XAS and STM to study the shape of spectra at the edge of Fe (II) L_2,3_ and the homogeneity of the film on different substrates. For all substrates at low temperature, the spectra never showed a pure LS state for the thin film, in contrast with the bulk material. A mixture of LS and HS was observed at 4 K and a pure HS at 290 K. This spin state mixture on a metallic substrate could be due to the epitaxial constraint imposed by the substrate on the molecular layer. STM revealed that the spin state mixture is not due to the inhomogeneity of thin films on the substrate, but rather it is due to the metal–molecule interface. They also observed the LS state increases after light illumination with samples on Cu(111) and Au(111), but no such behavior was reported on Ag(111).

Beyond the discussed factors that can affect SCO film functionality, there are other aspects to consider. These include oxidation, surface packing effects, and changes in coordination [154]. For thin films deposited via drop casting, the choice of solvent used can drastically alter SCO properties [139,155]. Molecule size, surface morphology [156,157], size reduction effects [156,158,159], and the interactions between microcrystals can all have a major effect on SCO properties [160]. Post-deposition treatments, such as solvent vapor annealing are a way to improve crystallinity and consequently the properties of the deposited films [161].

## 7. Insights from Theory

In this section, we focus on computational and analytical methods applied to study the interaction between SCO/substrate. While evidence gathered by direct observation using tools such as STM or XAS can give insight into the behavior of SCO molecules on different substrates, computational tools can provide a unique and novel perspective, especially in the case where it is difficult to explain a phenomenon by experiment alone. For some common substrates that were studied experimentally, parallel theoretical works led to some intriguing results. Density functional theory (DFT) calculations have been used to predict the initial spin state of different SCO complexes in different surface environments [114].

**Figure 20:**
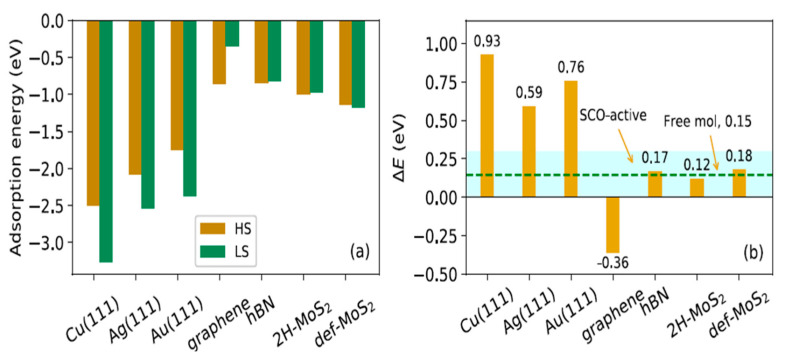
(**a**) The adsorption energies of Fe phen molecules for both the HS and LS states deposited on different substrates: Cu(111), Ag(111), Au(111), graphene, hBN, 2H–MoS_2_, and MoS_2_ with defects (def-MoS_2_). (**b**) The correlating spin adiabatic energy difference ΔE of the adsorbed molecule compared to the free molecule. The highlighted area corresponds to the range of ΔE (0–0.3 eV) for most SCO-active compounds. Adapted with permission from reference [104].

Both simulations and experiments show that 2D materials can serve as effective substrates that preserve the switching behavior of SCO molecules [25,57,104,162]. Zhang et al. [104] studied the surface effect on the switching mechanism of [Fe(phen)_2_)(NCS)_2_] complex on different metallic and 2D substrates using DFT. Calculations show that the LS state of [Fe(phen)_2_)(NCS)_2_] was locked on Cu(111), Ag(111), and Au(111) metallic substrates due to conformation changes in the adsorbate [104]. Most likely, due to the strong chemical interactions of SCO molecules with metal surfaces, metallic substrates cause a locking of the spin state. STM studies showed that the NCS group of [Fe(phen)_2_)(NCS)_2_] easily adsorbs into Cu substrates and causes a lock in the spin state [73]. Calculated adsorption energies of [Fe(phen)_2_)(NCS)_2_] for both HS and LS states for Cu substrates and others show that the spin state energy is very sensitive to the SCO-substrate interaction upon spin conversion. In the case of metallic substrates like Cu(111), Ag(111), and Au(111), based on Figure 20, the adsorption energies in the LS state are significantly lower compared to those in the HS state, which can cause the molecules to become locked in a LS ground state. DFT calculations showed that the [Fe(phen)_2_)(NCS)_2_] SCO molecule is preserved on both hexagonal boron nitride and molybdenum disulfide (MoS_2_), and the LS states were locked on Cu(111), Ag(111), and Au(111). On the contrary, [Fe(phen)_2_)(NCS)_2_] in contact with a graphite substrate exhibits a HS ground state. Calculations show that the spin transition temperature depends critically on surface environments correlated with the modification of electronic structures and molecular vibrations upon adsorption [104].

The Van der Waals interaction is the dominating force with 2D materials, which causes the differences between energies in LS and HS states not to vary much. With graphene, however, this does not hold true. It might explain why very few pieces of literature report a locked spin state when graphite is used as a substrate [26]. For the substrate 2H-MoS_2_, the difference between energies in the HS and LS states is less than 0.03 eV compared to the free molecule, which means an easy thermal spin switching is expected. This agrees with experimental results from MoS_2_/SCO, indicating that the sublimated SCO molecules on a MoS_2_ surface preserve their functionality [25]. Using a nanosheet hBN and MoS_2_ helps spin transition happen and even a single sulfur vacancy in MoS_2_ can shift the transition temperature toward higher values in comparison with a perfect surface.

**Figure 21:**
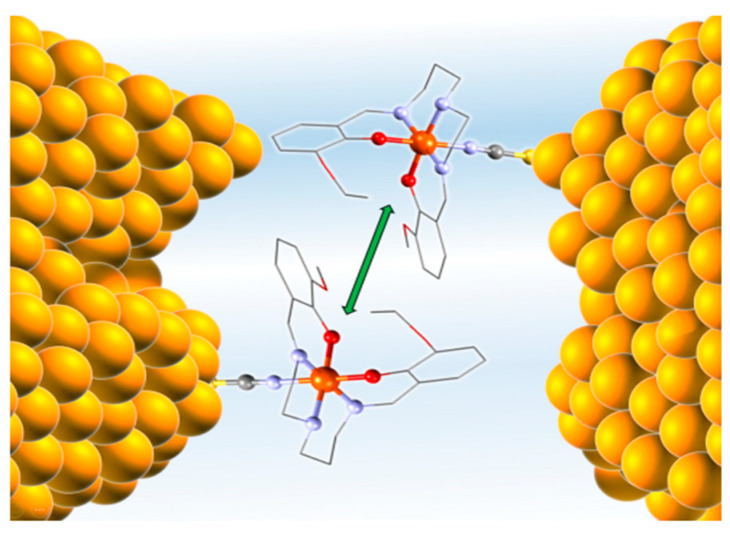
An image of the system: Two coupled [Fe^III^(EtOSalPet)(NCS)] SCO molecules situated in the space between two gold electrodes. Adapted with permission from reference [117].

Some experimental studies suggest that a nano-gap gold electrode device will not lock the spin state of SCO molecules [8,48,115,116,163] but this tends to contradict the observation that gold substrates in fact lock the spin state, as discussed above. Conductance calculations for a single SCO molecule between gold electrodes suggest that the spin state is dependent on the orientation of the SCO molecule [164]. Single-molecule conductance measurements of [Fe^III^(EtOSalPet)(NCS)] in the nanogap between gold electrodes demonstrate that switching occurs at higher temperatures compared to the bulk form due to a cooperative switching effect loss [163]. However, statistical analysis of the experimental resistance values, the occupation probabilities, and the lifetimes of the respective spin states revealed SCO molecules do not act independently and in fact couple to one another [117]. Although coupling between SCO molecules in such devices does not directly deal with the electrode, the way gold electrodes cause molecules to align in the gap indirectly influences the coupling between SCO molecules. Figure 21 is a schematic picture of this showing how [Fe^III^(EtOSalPet)(NCS)] SCO molecules tend to bond to the Au surface which leads to a coupling between molecules and consequently affects the spin state. Such single-molecule conductance calculations are often somewhat suspect because in many cases there is no locking of the spin state by the gold, and the calculated currents are high, incompatible with the high resistance of most spin crossover complexes suggestive of unrealistically high power dissipation through a single molecule.

Different models such as Ising-like or Slichter-Drickamer mean-field approach were used to study the effect of cooperativity, both qualitatively and quantitatively [165,166,167,168,169,170]. Moreover, Monte Carlo simulations based on a 3D mechanoelastic model provided more detailed and precise features of thermal transition for nucleation-propagation of like-spin domains of SCO systems, and for SCO molecules deposited on deformable substrates [171].

**Figure 22:**
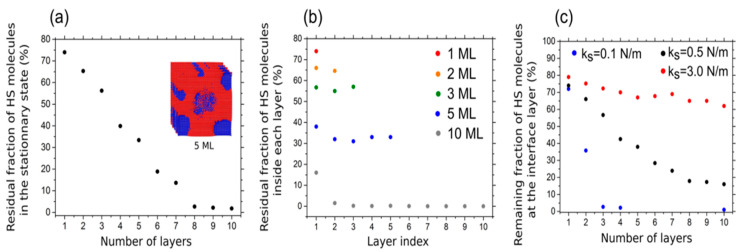
Monte Carlo Arrhenius calculations of [Fe^II^((3,5-(CH_3_)_2_Pz)_3_BH)_2_] molecules for (**a**) the residual fraction of high-spin (HS) molecules as a function of the number of layers for k_s_ = 0.5 N·m^−1^. Inset: Image of the residual fraction of HS molecules for the 5 molecular layers system. (**b**) The division of the residual fraction of HS molecules for each layer. Red, 1 molecular layers; orange, 2 molecular layers; green, 3 molecular layers; blue, 5 molecular layers; gray, 10 molecular layers. (**c**) The progression of the remaining fraction of HS molecules in the intersecting layer as a function of the total number of layers. Blue, k_s_ = 0.1 N·m^−1^; black, k_s_ = 0.5 N·m^−1^; red, k_s_ = 3.0 N·m^−1^. Adapted with permission from reference [75].

Monte Carlo simulations can help predict the behavior of the SCO molecules at the interface of different substrates and model how the thickness change can potentially modify the SCO transition temperature of the [Fe^II^((3,5-(CH_3_)_2_pz)_3_BH)_2_] complex [75]. Simulations show that both film thickness and substrate play a crucial role in the spin state of [Fe^II^((3,5-(CH_3_)_2_pz)_3_BH)_2_]. Figure 22a shows that by increasing the number of molecular layers, the HS fraction of [Fe^II^((3,5-(CH_3_)_2_pz)_3_BH)_2_] sublimated on a Cu(111) substrate decreases, and for more than 8 molecular layers of SCO, the LS spin state is predicted. Figure 22b describes the proportion of molecules in the HS state decomposed in different layers of film to Figure out how the HS state is distributed in different layers. Based on the simulations, the HS state is dominant due to the epitaxial constraint forced by the stiffness constant, however, the interfacial HS proportion decreases by increasing the number of layers. This implies that the total HS state fraction is a combination of the interfacial constraint and the cooperativity forced by the other layers. Figure 22c shows that different stiffness constants significantly alter the HS state fraction of the film on the interface of a metallic surface. For the low stiffness constant (k_s_), a complete transition of the interface layer for a three molecular layer film is the result, however, for larger k_s_ values, the HS state fraction on the first layer does not dramatically decrease by increasing the thickness as the molecule–substrate interaction dominates, and the molecular interlayer interaction becomes negligible. This study revealed that for a strong interaction between the SCO complex and substrate, the HS state alters with increasing the film thickness.

Beyond this, computational studies explain that mixed-state domains can form due to extensive intermolecular interactions [172] which introduce another parameter that affects the functionality of SCO complexes beyond SCO–substrate interaction. Ab initio calculations verified that adsorption of Fe(-phen)_2_(NCS)_2_ on CuN results in less than half of the value compared to Cu for both HS and LS states which leads to an easier switching to the other spin state [79] confirming previous experimental results [71].

Although experimental works show oxidized magnets lock the spin state of SCO molecules [103], computational studies of Fe(1,10-phenanthroline)_2_(NCS)_2_ (Fephen), using DFT, suggest that some ferromagnetic substrates like cobalt reduce the SCO-substrate interaction at the interface compared to substrates like Au and Cu. This eases the spin state switching significantly and might be due to an indirect exchange mechanism (magnetic coupling) between the Fe atom in the center and Fe-phen and the Co substrate. The Fephen/substrate interface remains active magnetically due to the presence of magnetic moments in the NCS group and the center Fe atom [173]. Computational studies on the effect of other layers like AlN deposited on an Al(100) substrate for this Fe-phen molecule show that [80] the splitting energy between HS and LS states slightly increases once deposited on Al(100) and tends to be locked into the LS state, as already observed in some experimental works [85,88]. A coating layer of AlN reduces the splitting energy as it promotes molecular adsorption, leading to an easier switching between two spin states [80].

**Figure 23:**
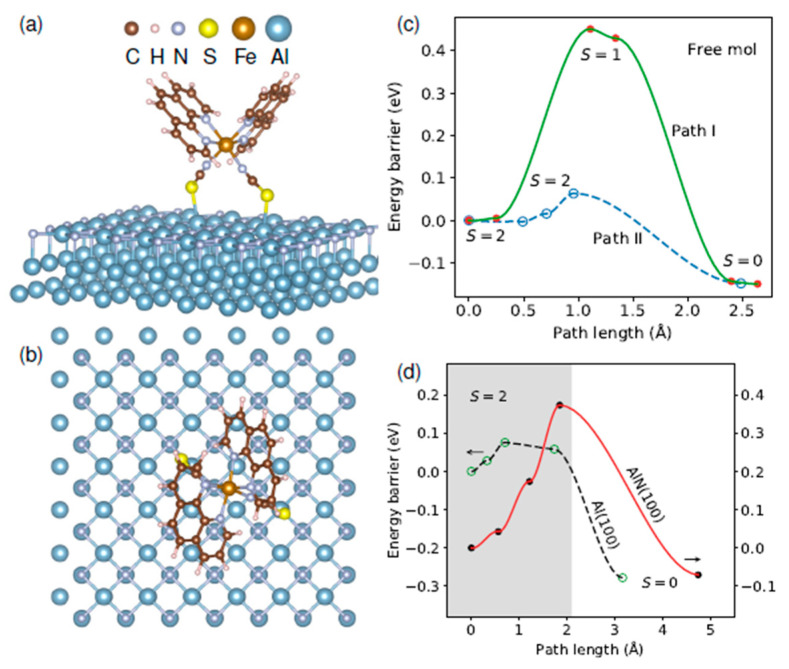
(**a**) The side and (**b**) top view of the Fe-phen molecule deposited on a surface of AlN(100). The GGA+U calculated spin transition barrier following the minimum energy path (MEP) between HS (S = 2) and LS (S = 0) states for (**c**) the free molecule and (**d**) the adsorbed molecule on Al(100) and AlN(100) substrates, acquired from GGA+U. The MEP is ascertained from structural relaxations with a constraint on S-S distance and the angle between NCS groups. For the free Fe-phen molecule, two paths are studied: the one including the intermediate-spin state (S = 1) (path I) and the one displaying a direct HS-LS spin relaxation (path II). For the deposited molecule, only path II is studied. Adapted with permission from reference [80].

Figure 23a,b show a single Fe-phen molecule on the AlN interface. In Figure 23c two different paths are studied for the free molecule Fe-phen: one involving an intermediate spin state, S = 1 (path I), and one with a direct phase transition from HS to LS (path II). The data plots illustrate that path II is more likely to happen [80]. So, by selecting path II and comparing the energy paths along with the spin transition of a single-molecule Fephen deposited on Al(100) and AlN(100) (Figure 23d), the spin transition barriers increased for both molecules on surfaces compared to the free molecule; however, the HS to LS barrier on Al(100) is only slightly altered (by 0.01 eV). The energy barrier of the reverse process is significantly increased (by 0.14 eV). This change in SCO barriers is outstanding in the case of AlN(100) where the energy barrier is increased by 0.30 eV from HS to LS and 0.09 eV for the reverse spin transition.

**Figure 24:**
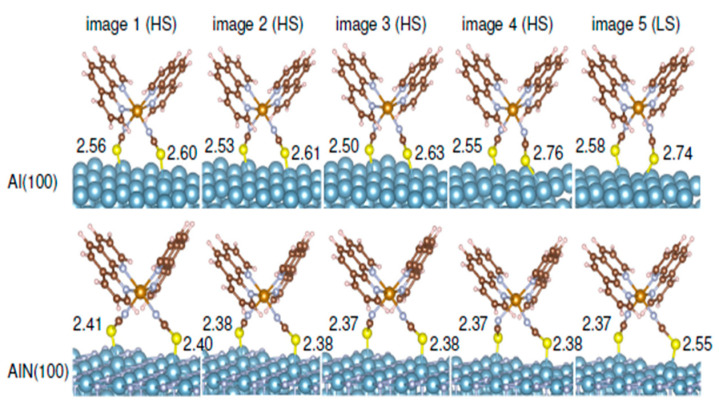
The molecular diagrams of the images following the minimum energy path for the HS-LS transition acquired by constraint structural relaxations using GGA+U. The S-Al, nearest Al atom, and distances (in Å) are shown next to each image. Adapted with permission from reference [80].

Molecular configurations along the minimum energy path of the Fe-phen complex on Al and AlN (Figure 24) show that although the structural properties related to the NCS groups may be modified on different substrates, as compared to the free molecule, the Fe-N distances are not altered dramatically. Calculations show that the decrease of S–S distances from HS to LS states is significantly larger than that of the free molecule. Also, the N-Fe-N angle will not decrease monotonically for molecules on the substrate (mostly AlN) unlike in the isolated molecule. Different from Al(100), with the AlN(100) surface, the Al-S bond distances are decreased, evidence of a stronger chemical interaction at the interface. Structural relaxations indicate that the Al atoms in contact with S atoms tend to lift up and out of the substrate to enable molecule-surface coupling. The potential barriers between phase transitions at the surface seem to be the result of molecular adsorptions. With changes in the volume of the molecule that occur during phase transition, the adsorption sites change. It may be that the energy barrier becomes higher in the case of A1N(100) due to the larger energy required to move the NCS groups.

The energy barrier of surface-enhanced spin transition might result in a wider thermal hysteresis loop. The thermal hysteresis of [FeH_2_B(pz)_2_(bipy)] was demonstrated to be more pronounced in thinner films deposited on the Al_2_O_3_ substrate [132]. This implies that the cooperative effects, generating the hysteresis, are significantly influenced by molecule-surface interactions, which correspond with the SCO energy barrier.

The DFT calculations of [Fe(tzpy)_2_(NCS)_2_] complex deposited on an Au(100) substrate [174] reveal three important points: First, the complex can be adsorbed on any of the three different sites with similar energies on the surface, however, the bridge coordination is energetically a preference, which is seen for other SCO molecules deposited on metallic substrates. Second, deposition of [Fe(tzpy)_2_(NCS)_2_] on Au substrates increases the splitting energy between HS and LS states by around 15–25%, leading to the SCO molecule on the interface stabilizing in the LS state in agreement with similar experimental and computational studies [85,86,175]. And finally, the spin state of the deposited SCO complex on the substrate can be predicted by simulating a bias voltage of the STM tip, indicating at low temperatures that the [Fe(tzpy)_2_(NCS)_2_] complex coexists in both HS and LS spin states, as already seen for similar SCO molecules with Fe centers deposited on Au substrates [85,86,88].

## 8. Conclusions

There has been much progress in better understanding the various issues related to the SCO–substrate interaction, but more guidance from theory is essential as there is still much to be understood about the physical processes involved in SCO–substrate interactions. Metals can suppress spin state changes due to their robust interaction with at least a few deposited SCO molecular layers. While metallic substrates tend to cause spin state locking, both experimental and computational studies verify that 2D substrates with a lower density of states near the Fermi level are a far better candidate for use in SCO molecular-based devices. Our review of the literature seems to indicate that among the various substrates investigated thus far, as a substrate, graphene generally does not perturb the spin state or spin state changes of an adsorbed SCO molecular layer. So, while the free electron density may play a significant role in spin state locking, as suggested by sections two and three, in the meantime, graphene is a gapless semiconductor with a very low density of states at the Fermi level. While most studies of SCO molecules with 2D materials have focused on the graphene–SCO interfaces, new experimental and theoretical efforts have shown that different types of 2D semiconductor materials, such as MoS_2_, WSe_2_, and HfS_2_ might be promising [25,57,104]. Ferroelectric polarization of the substrate can favor one spin state over another so under some conditions, spin state in a SCO film can be manipulated by changing the polarity of the ferroelectric substrate. Just the same, there remain many fundamental questions that need to be addressed if there is to be a practical route to scalable devices. There are multiple contributions to the total magnetic moment and magnetic anisotropy yet to be fully explored theoretically and experimentally, while estimates of the interaction strength and the nature of the interaction between SCO molecules and substrates remain in their infancy. While more insight from theory is vital to gaining a better understanding of the interaction between SCO molecular thin films and various substrates, surface morphology and the details of the structure are needed as well for the theory to proceed.

## Data Availability

Not applicable.
